# PIWI-Interacting RNAs (piRNAs): Promising Applications as Emerging Biomarkers for Digestive System Cancer

**DOI:** 10.3389/fmolb.2022.848105

**Published:** 2022-01-27

**Authors:** Aiting Cai, Yuhao Hu, Zhou Zhou, Qianyi Qi, Yixuan Wu, Peixin Dong, Lin Chen, Feng Wang

**Affiliations:** ^1^ Department of Laboratory Medicine, Affiliated Hospital of Nantong University, Nantong, China; ^2^ Department of Obstetrics and Gynecology, Hokkaido University School of Medicine, Hokkaido University, Sapporo, Japan; ^3^ Department of Gastroenterology and Laboratory Medicine, Nantong Third Hospital Affiliated to Nantong University, Nantong, China

**Keywords:** Piwi-interacting RNA, cancer biomarker, diagnosis, prognosis, digestive system cancer, therapeutic target

## Abstract

PIWI-interacting RNAs (piRNAs) are a novel type of small non-coding RNAs (sncRNAs), which are 26–31 nucleotides in length and bind to PIWI proteins. Although piRNAs were originally discovered in germline cells and are thought to be essential regulators for germline preservation, they can also influence gene expression in somatic cells. An increasing amount of data has shown that the dysregulation of piRNAs can both promote and repress the emergence and progression of human cancers through DNA methylation, transcriptional silencing, mRNA turnover, and translational control. Digestive cancers are currently a major cause of cancer deaths worldwide. piRNAs control the expression of essential genes and pathways associated with digestive cancer progression and have been reported as possible biomarkers for the diagnosis and treatment of digestive cancer. Here, we highlight recent advances in understanding the involvement of piRNAs, as well as potential diagnostic and therapeutic applications of piRNAs in various digestive cancers.

## 1 Introduction

Cancer is the leading cause of death and a serious public health problem in China (Zeng et al., 2018; Feng et al., 2019). In 2018, half of the newly diagnosed cancers in China were in the digestive system (Zeng et al., 2018; Feng et al., 2019). More than one-third of all deaths were related to the digestive tract (Zeng et al., 2018; Feng et al., 2019). According to the Global Cancer Burden report, three of the top five most common cancers are digestive system cancers: hepatocellular carcinoma (HCC), gastric cancer (GC), and colorectal cancer (CRC) (Bray et al., 2018). Therefore, timely detection and standardized treatment are particularly critical. Studies have confirmed the key role of non-coding RNAs (ncRNAs) in mediating human carcinogenesis (ENCODE Project Consortium, 2012). PIWI-interacting RNAs (piRNAs) are the least studied sncRNAs and participate in epigenetic and retrotransposon post-transcriptional gene silencing by interacting with PIWI proteins (Xiao and Ke, 2016; Ozata et al., 2019). piRNAs were first identified in germ cell lines and their expression was also confirmed in somatic tissues (Girard et al., 2006; Martinez et al., 2015). piRNA precursors are transcribed from piRNA clusters, modified in the cytoplasm, and transported into the nucleus, where piRNAs form complexes with PIWI proteins (Zhang et al., 2018). Some studies have shown that abnormally expressed piRNAs are closely related to a variety of malignancies (Yin and Lin, 2007; Ku and Lin, 2014). This article focuses on the regulatory role of piRNAs and PIWIs in digestive system cancers and discusses the potential clinical applications of piRNAs in digestive cancer diagnosis and treatment.

## 2 Origin and Function of PIWI-Interacting RNAs

### 2.1 PIWI-Interacting RNAs and PIWI

piRNAs have the following six characteristics: 1) piRNAs are approximately 26–31 nucleotides in length, whereas microRNAs and siRNAs have lengths of 21–23 nucleotides. piRNAs are independent of the Dicer enzyme and are produced by a single-stranded precursor (Weng et al., 2019). 2) The majority of piRNA clusters in somatic cells are unidirectional, whereas the majority of germline piRNA clusters are dual-stranded (Yamanaka et al., 2014). 3) The majority of mature primary piRNAs contain uridine at the 5′ end, and the 3′ ends of piRNAs are uniquely methylated 2-OH structures (Hirakata and Siomi, 2016). 4) piRNAs are unevenly distributed among various genomic sequences, including exons, introns, and repeat sequences (Aravin et al., 2006; Girard et al., 2006; Grivna et al., 2006). 5) piRNAs are derived not only from the transposons themselves but also from the flanking genomic sequences (Aravin et al., 2006; Girard et al., 2006; Grivna et al., 2006). 6) piRNAs are not degraded in circulation and are stably expressed in body fluids (Yang et al., 2015; Freedman et al., 2016).

piRNAs have been detected in somatic cells and germ cells of mammals (mice and humans), Drosophila (Kawamura et al., 2008), Caenorhabditis elegans (Batista et al., 2008), and zebrafish (Houwing et al., 2008). Argonaute proteins are divided into the AGO subfamily and PIWI subfamily. PIWI proteins are mainly expressed in the germline and human tumors (Höck and Meister, 2008). The human PIWI protein subfamily consists of PIWIL1, PIWIL2, PIWIL3 and PIWIL4 (Höck and Meister, 2008). piRNAs are essential in many stages of spermatogenesis, and PIWIs are necessary to maintain the function of reproductive system stem cells (Weng et al., 2019). The absence of piRNAs can lead to pathogenic effects in the reproductive system, such as birth defects and infertility (Weng et al., 2019).

### 2.2 Biological Formation of PIWI-Interacting RNAs

piRNAs can be classified into three derived sources: lncRNAs, mRNAs, and transposons (Cheng et al., 2019). Most in-depth research has focused on the transposon source of piRNAs. piRNAs are produced from single-stranded precursors, and Dicer enzymes are not required. piRNA biogenesis has little in common with siRNA and miRNA biogenesis (Cheng et al., 2019). The biogenesis of piRNAs involves two pathways: primary amplification and secondary amplification (also described as a ping-pong amplification loop) (Cheng et al., 2019).

Several proteins, including RNA polymerase II, the Rhino- Deadlock- Cutoff complex (RDC complex), Moonshiner (Moon), TATA-box binding protein (TBP)-related factor 2 (TRF2), three prime repair exonuclease (TREX), and 56-kDa U2AF-associated protein (UAP56), are involved in the transcription of piRNA precursors in the nucleus (Aravin et al., 2006; Girard et al., 2006; Weng et al., 2019; Wu et al., 2020). RNA polymerase II is first recruited to piRNA clusters, and the RDC complex then helps to promote transcription. Moon interacts with the RDC complex and TRF2 to enhance transcription start. TREX prevents R-loop formation, and UAP56 inhibits dual-strand cluster splicing. After nuclear transport, piRNA precursors are resolved by the RNA helicase Armitage (Armi), and precursors are processed into pre-piRNAs by the endonuclease Zucchini (Zuc). Then, pre-piRNAs are loaded onto the PIWI proteins (PIWI and Aubergine), trimmed by an exonuclease Nibbler and methylated by the Hen1 methyltransferase.

In secondary amplification, primary piRNAs are stimulated through the catalysis of the AGO3 and Aubergine (Aub) proteins, finally producing mature piRNAs (Wu et al., 2020). Aub is loaded with piRNAs and this complex recognizes and cleaves complementary RNAs (such as transposon mRNAs or transcripts derived from the opposite strand of the same piRNA cluster). This cleavage produces the 5′ end of a new piRNA, which is subsequently loaded into AGO3 and induces the cleavage of complementary RNA. This results in a new piRNA that is identical in sequence to the piRNA that initiated the cycle (Wu et al., 2020). With repeated cutting, piRNA production is amplified. Therefore, generating a large number of piRNAs in a short time is called the ping-pong loop (Zhang et al., 2011). The piRNAs generated by the ping-pong loop are mature piRNAs. Once mature piRNAs or piRNA/PIWI protein complexes are formed, they can bind to target genes in the nucleus to silence or delay target gene transcription (Luteijn and Ketting, 2013).

### 2.3 Biological Functions of PIWI-Interacting RNAs

#### 2.3.1 piRNAs and Transposon Silencing

In piRNA biogenesis, piRNA clusters are located in transposon elements. Thus, piRNAs are thought to be involved in transposon silencing through epigenetic mechanisms (DiGiacomo et al., 2013). Transposable elements shift and replicate by inserting themselves into the genome (Tóth et al., 2016). Improper insertion of transposable elements may lead to genomic mutations, such as chromosome deletion, duplication, and rearrangement (Hedges and Deininger, 2007). The activation of transposable elements will affect the integrity of the genome, which is very important for the transmission of genetic information. The activation of transposable elements can also damage DNA and lead to meiosis arrest, which in turn affects the growth and development of stem cells. The over-activation of transposable elements is potentially highly pathogenic and is quite harmful to the organisms (Vagin et al., 2006). piRNAs maintain genomic integrity by silencing transposons (Fu and Wang, 2014; Lin et al., 2021). It has been proved that piRNAs interact with PIWI subfamily proteins, resulting in the development of the piRNA-induced silencing complex (piRISC), which detects and silences complementary sequences at the transcriptional (TGS) and post-transcriptional (PTGS) levels (Czech et al., 2018; Cheng et al., 2019). In the TGS, gene expression is suppressed by altering the chromosome. PTGS works through mRNA destabilization and mRNA translation inhibition (Liu et al., 2004; Phay et al., 2018).

#### 2.3.2 PIWI-Interacting RNAs and DNA Methylation

DNA methylation is a type of DNA chemical modification and refers to the process of selectively adding S-Adenosyl-l-methionine (SAM) to specific bases by DNA methyltransferase (DNMT) (Pan et al., 2018). In the piRNA-PIWIL1 pathway, the activation of PIWIL1 can lead to a global loss of hypomethylation and specific regional changes in hypermethylation (Litwin et al., 2017). Hypomethylation can promote mitotic recombination and lead to chromosome deletion, ectopic rearrangement, and rearrangement (Sciamanna et al., 2011). Hypermethylation mostly occurs in the CpG islands of the promoter region. Under the regulation of DNMT, tumor suppressor genes can be inactivated, and transcription can be suppressed. The PIWI-piRNA pathway contributes to tumorigenesis through this mechanism (Yan et al., 2015). DNA methylation is also a critical mechanism leading to transposon silencing (Weng et al., 2019).

#### 2.3.3 PIWI-Interacting RNAs and mRNA

After transcription, piRNAs have a function similar to that of microRNAs. They can induce mRNA degradation (Pek et al., 2012; Yu et al., 2019), thus hindering protein synthesis (Dai et al., 2020). The piRNA-mediated mRNA degradation can occur through two major mechanisms: either by the slicing of mRNA by PIWI or via a deadenylation-dependent mechanism (Rouget et al., 2010; Zhang et al., 2015). piRNAs and microRNAs are both important non-coding small RNAs, and they regulate gene expression. Whether piRNAs have a function similar to that of microRNAs requires further investigation.

#### 2.3.4 Workflow for PIWI-Interacting RNA Discovery and Analysis

**FIGURE 1:**
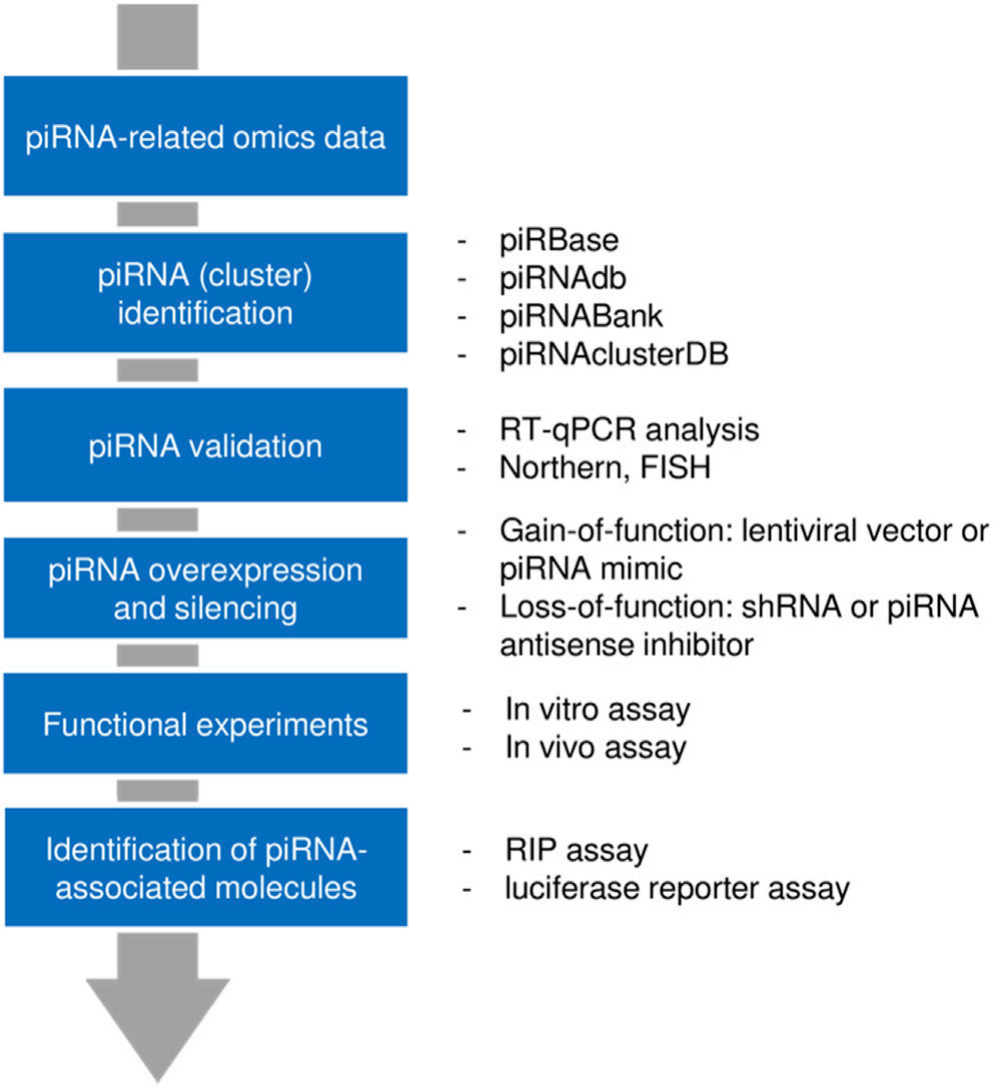
Workflow for piRNA discovery and analysis. First, a candidate piRNA was identified from a pool of RNAs through high-throughput approaches. After validation, gain-of-function or loss-of-function models were generated and the functional impact of a piRNA was assessed. By using pulldown assay or reporter assay, those molecules that interact with piRNAs (proteins or RNAs) could be determined.

In general, a piRNA of interest can be extracted from non-piRNA molecules using high-throughput approaches (such as RNA-sequencing or microarray analysis). Multiple piRNA databases have been established for piRNA annotation. Northern blotting, *in situ* hybridization, and reverse transcription-quantitative polymerase chain reaction (RT-qPCR) are frequently used for experimental validation of piRNAs. The functional effect of a circRNA can be examined after piRNA silencing with shRNA or piRNA antisense inhibitor, or lentiviral vector/piRNA mimic-mediated piRNA overexpression. *In vitro* and *in vivo* assays provide essential insights into the piRNA’s function in tumor cells. Finally, the molecules interacting with the piRNA (proteins and RNAs) could be identified using RNA binding protein immune-precipitation (RIP) experiments and luciferase reporter assays, respectively (Cheng et al., 2019; Huang et al., 2021; Liu et al., 2021) (Figure 1).

## 3 PIWI-Interacting RNAs in Digestive System Cancers

The dysregulation of piRNA expression has been associated with various diseases, especially tumors and reproductive system diseases (Liu et al., 2019). piRNAs have pro-cancer or anti-cancer functions in cancer initiation, progression, and metastasis (Guo et al., 2020). piRNAs not only affect the growth, apoptosis, and invasion of tumor cells but also control cancer cell metastasis (Liu et al., 2018; Shen et al., 2018). In breast cancer, the levels of piR-4987 are positively correlated with lymph node metastasis (Huang et al., 2013). In addition, piR-823 expression is 2-fold higher in poorly differentiated colorectal cancer (CRC) tissues than in well/moderately-differentiated CRC tissues (Sabbah et al., 2021). The upregulation of piR-823 is associated with the presence of distant metastasis in gastric cancer (GC) patients (Cui et al., 2011).

**FIGURE 2:**
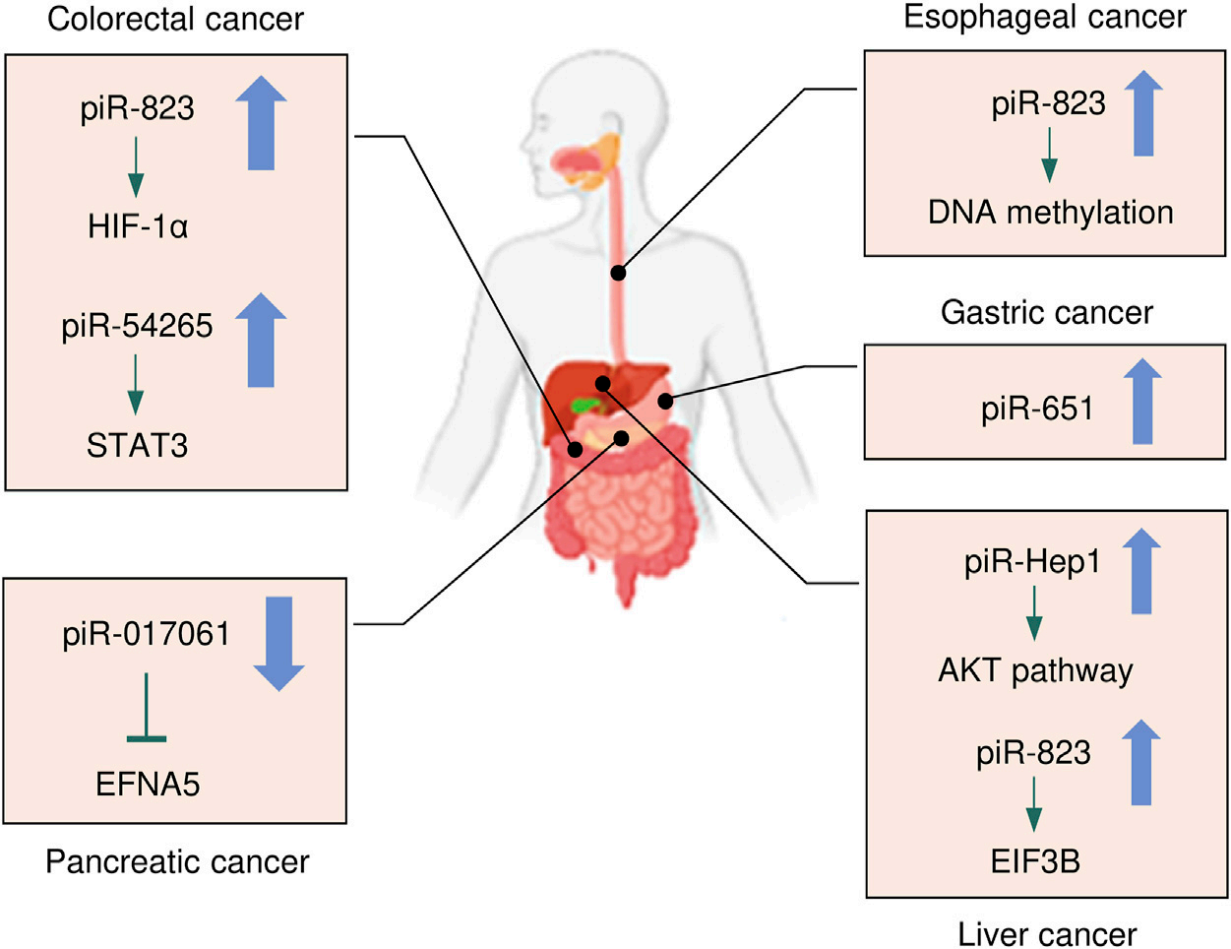
The expression and underlying mechanisms of piRNAs in digestive system cancers. Oncogenic and anti-oncogenic piRNAs and their influence on the downstream pathways in digestive system cancers are shown. The up arrow indicates oncogenic piRNAs that are upregulated in digestive system cancers, while the down arrow suggests tumor-suppressive piRNAs that are downregulated in digestive system cancers.

**FIGURE 3:**
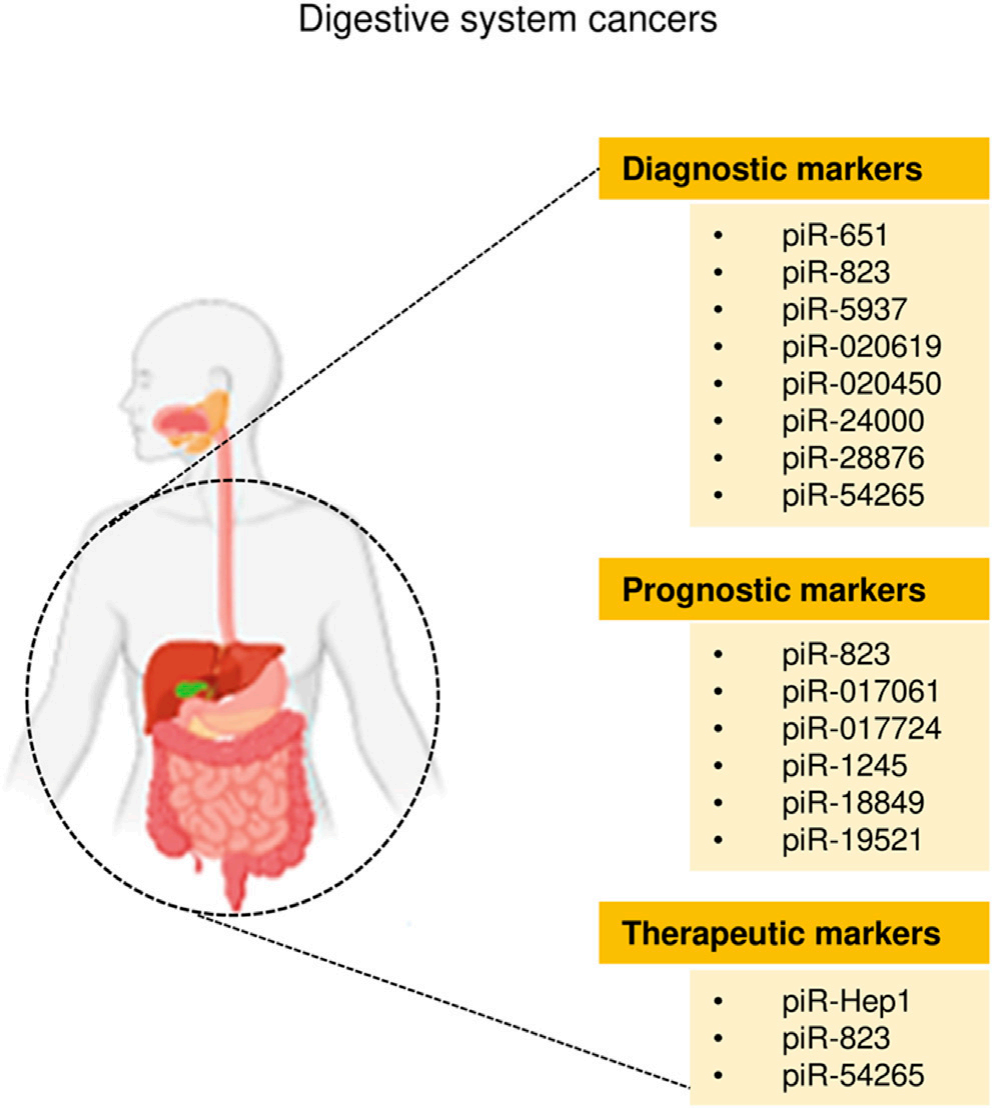
The clinical application of piRNAs in digestive system cancers.

Cancers of the digestive system include HCC, GC, CRC, pancreatic cancer, esophageal cancer, and biliary tract cancer. There is increasing evidence to support a strong association between piRNAs, PIWI proteins, and digestive system cancers (Cui et al., 2011; Sabbah et al., 2021). The aberrant piRNA expression affects the tumorigenesis and progression of digestive system cancers (Figures 2, 3). The current understanding of piRNAs and PIWI proteins in major digestive system cancers has been summarized in Table 1.

**TABLE 1:** Summary of piRNAs and PIWI proteins in digestive system cancers.

Cancer type	piRNA	Expression	Biomarker utility	Source	Detection method	Ref
HCC	piR-Hep1	Upregulation	Promotes cell viability, motility, invasiveness, and activates the AKT pathway; therapeutic target	Cell lines, tissue	RT-qPCR, RNA sequencing, northern blotting	Law et al. (2013)
	PIWIL2/PIWIL4	Upregulation	Prognostic biomarker	Tissue	Tissue chips, immunofluorescence staining	Zeng et al. (2017)
Hepatic fibrosis	piR-823	Upregulation	Binds to EIF3B to activate HSCs via upregulating TGF-β1	Activated HSCs	RT-qPCR, CCK-8, BrdU, RNA pull-down, liquid chromatography-mass spectrometry assay	Tang et al. (2018)
CRC	piR-017724	Downregulation	Prognostic biomarker	Tissue	RT-qPCR, RNA sequencing	Qu et al. (2019)
	piR-18849	Upregulation	Prognostic biomarker; positively correlated with lymph node metastasis and tumor grade	Tissue	RT-qPCR, RNA sequencing	Yin et al. (2019)
	piR-19521	Upregulation	Prognostic biomarker; negatively correlates with the degree of tumor differentiation	Tissue	RT-qPCR, RNA sequencing	Yin et al. (2019)
	PIWIL1	Upregulation	Prognostic biomarker; correlates with tumor differentiation degree, infiltration depth, lymphovascular invasion, lymph node metastasis and TNM stage	Tissue	Kaplan-Meier method, Cox’s proportional hazards model, IHC and RT-qPCR	Sun et al. (2017)
	piR-5937	Downregulation	Diagnostic biomarker; decreased with advanced clinical stage	Blood serum	RT-qPCR	Vychytilova-Faltejskova et al. (2018)
	piR-28876	Downregulation	Diagnostic biomarker; decreased with advanced clinical stage	Blood serum	RT-qPCR	Vychytilova-Faltejskova et al. (2018)
	piR-020619	Upregulation	Diagnostic biomarker	Serum	RT-qPCR, ROC curve analysis	Wang et al. (2020)
	piR-020450	Upregulation	Diagnostic biomarker	Serum	RT-qPCR, ROC curve analysis l	Wang et al. (2020)
	piR-823	Upregulation	Inhibits the ubiquitination of HIF-1α by up-regulating the G6PD, up-regulates the glucose consumption of carcinoma cells and inhibits intracellular ROS; prognostic and therapeutic biomarker	Cell lines, tissues	RT-qPCR, CCK-8, invasion, apoptosis, glucose consumption assay, detection of intracellular ROS and half-life of G6PD	Feng et al. (2020)
	piR-823	Upregulation	Upregulates phosphorylation and transcriptional activity of HSF1; therapeutic target	Cell lines, tissue	CCK-8, cell cycle, colony formation, apoptosis, luciferase reporter, RIP assay	Yin et al. (2017)
	piR-24000	Upregulation	Diagnostic biomarker	Tissue	RT-qPCR	Iyer et al. (2020)
	piR-54265	Upregulation	Forms PIWIL2/STAT3/ p-SRC complex to activate STAT3 signaling; therapeutic target	Cell line, tissue, animal	Cell viability, colony formation, apoptosis, invasion, migration, RIP assay, animal experiments	Mai et al. (2018)
	piR-54265	Upregulation	Diagnostic biomarker	Tissue, serum	RT-qPCR	Mai et al. (2020)
	piR-1245	Upregulation	Prognostic biomarker	Cell lines, tissue	MTT, colony formation, invasion, migration, apoptosis assay	Weng et al. (2018)
GC	PIWIL1/2	Upregulation	Prognostic biomarker	Tissue	IHC	Wang et al. (2012)
	piR-651	Upregulation	Diagnostic biomarker	Peripheral blood	RT-qPCR	Cui et al., 2011
	piR-823	Upregulation	Diagnostic biomarker	Peripheral blood	RT-qPCR	Cui et al., 2011
	piR-651	Upregulation	Inhibits cell proliferation; diagnostic biomarker	Cell lines, tissue	MTT assay, cell cycle analysis, RT-qPCR	Cheng et al. (2011)
	PIWIL1	Upregulation	Prognostic biomarker	Cell lines, tissue	Wound-healing, invasion, cell proliferation assay	Gao et al. (2018)
	piR-823	Downregulation	Therapeutic target	Cell lines, tissue,	MTT, tumorigenicity assay	Cheng et al. (2012)
Pancreatic cancer	piR-017061	Downregulation	Inhibits cancer cell growth; prognostic biomarker	Cell lines, Tissue	Cell viability, colony formation assay, RT-qPCR	Müller et al. (2015), Xie et al. (2021)
Esophageal squamous cell carcinoma	piR-823	Upregulation	Induce DNA methylation, diagnostic biomarker	Tissue	RT-qPCR	Su et al. (2020)
	PIWIL1	Upregulation	Prognostic biomarker	Tissue	RT-qPCR, western blot, IHC	He et al. (2009)
Cholangiocarcinoma and gallbladder carcinoma	Exosomal piRNAs		Diagnostic biomarker	Blood	Exosome separation and RNA isolation, RNA sequencing and mapping	Gu et al. (2020)

### 3.1 PIWI-Interacting RNAs and Hepatocellular Carcinoma

HCC is one of the most common malignancies worldwide and is the second leading cause of death in men (Islami et al., 2017). Chronic infection accounts for more than 78% of liver cancer cases in China (Islami et al., 2017). From 2013 to 2021, the incidence of liver cancer has increased in both men and women (Ryerson et al., 2016). With no obvious symptoms or characteristics in the early stage, the onset of liver cancer can go undetected. Most patients are already in the middle or late stage when they are first diagnosed. Therefore, it is particularly important to explore biomarkers that could be utilized in the early diagnosis and treatment of HCC.

Rizzo et al. (2016) applied small RNA sequencing technology to analyze piRNA expression patterns in different stages of liver disease. Changes in piRNA expression profiles can distinguish HCC tissue from liver cirrhosis (Rizzo et al., 2016). The Wilcoxon-Mann-Whitney test was used to evaluate the difference in piRNAs in various patterns of liver disease. The specific expression of piRNAs in tumors has been revealed. For example, piR-020498 is upregulated in high-grade dysplastic nodules and advanced HCC but is nearly undetectable in nodules of other degrees. Additionally, piR-013306 is overexpressed only in HCC. These results showed that piRNAs are involved in the progression of HCC and show specific expression in each stage (Table 2). The presence of piRNA molecules was detected in all samples of HCC, verifying the involvement of these piRNAs in liver carcinogenesis.

**TABLE 2:** Changes of piRNA expression during human liver carcinogenesis.

	Low-grade dysplastic nodules	High-grade dysplastic nodules	Early hepatocellular carcinoma	Progressed hepatocellular carcinoma
piR-001078, -001207, -001346, -017061, -017295, -019420, -020450	✓	✓	✓	✓
piR-001170, -016975, -017724, -019951, -020828, -020829	✓	✓	✓	✓
piR-020498	✗	✓	✓	✓
piR-013306	✗	✗	✗	✓

✓: Presence; ✗: Absence.

Currently, the specific mechanisms by which piRNAs act in HCC remain unclear. Previous studies have indicated that piRNAs, such as piR-Hep1 (Law et al., 2013) and piR-823 (Tang et al., 2018), are closely linked with the occurrence and development of HCC. A novel piRNA, piR-Hep1, was identified through large-scale parallel sequencing (Law et al., 2013). When compared to normal cells, HCC cells have a 12-fold higher expression of piR-Hep1 (Law et al., 2013). Silencing of piR-Hep1 inhibited the proliferation, migration, and invasion ability of HCC cells (Law et al., 2013). Downregulation of piR-Hep1 also reduced the level of AKT phosphorylation (Nakanishi et al., 2005; Whittaker et al., 2010; Law et al., 2013). Interestingly, the expression of PIWIL2 was positively correlated with the level of piR-Hep1 in HCC tissues, implying that piR-Hep1 might mediate the PI3K/AKT pathway by binding to PIWIL2, thus playing a role in the function of HCC recurrence and progression. Hence, piR-Hep1 may represent a new therapeutic target for HCC.

The expression of piR-823 is significantly upregulated in activated hepatic stellate cells (HSCs), and the overexpression of piR-823 can promote HSC proliferation and the production of α-SMA and COL1a1. The binding of piR-823 with eukaryotic initiation factor 3B (EIF3B) activates HSCs in liver fibrogenesis by increasing transforming growth factor-β1 (TGF-β1) (Tang et al., 2018). Therefore, blockade of piR-823 might be a new strategy to treat liver fibrosis, a major risk factor for HCC.

The role of piRNAs is affected and regulated by their binding protein (Ding et al., 2018). RNA-binding proteins are also inextricably linked with HCC. Li et al. (2020) found that RNA-binding proteins help transform the physiological microenvironment into the tumor microenvironment by regulating protein synthesis, thus initiating the biogenesis of secondary mouse HCC. PIWIL1 (also known as HIWI) is a member of the PIWI subfamily. Studies have shown that PIWIL1 is highly expressed in HCC tissue and HCC cells (MHCC97L and MHCC97H) (Xie et al., 2015). The downregulation of PIWIL1, mediated by shRNA, restrains the proliferation and migration of HCC cells (Xie et al., 2015). The expression of PIWIL1 was positively associated with HCC tumor size and metastasis and negatively associated with the survival rate (Zhao et al., 2012). After the knockdown of PIWIL1, the proliferation, invasion, and metastasis of HCC cells were suppressed (Zhao et al., 2012). Therefore, PIWIL1 may be a latent biomarker or therapeutic target for HCC. Zeng et al. (2017) investigated the cellular localization and expression of the molecular chaperones PIWIL2 and PIWIL4. The authors found that the co-expression of PIWIL2 and PIWIL4 could be employed as an indicator of poor prognosis and malignancy in HCC. The above findings indicated that both piRNAs and PIWI proteins are associated with the occurrence and development of HCC, and they have the potential to be used as novel biomarkers for HCC (Figures 2, 3).

### 3.2 PIWI-Interacting RNAs and Colorectal Cancer

CRC has the third-highest cancer incidence and second-highest cancer mortality worldwide. It is among the top five mortality-causing cancers worldwide (Bray et al., 2018). The incidence of CRC has significantly increased in recent years (Chen et al., 2016; Bray et al., 2018). The detection efficiency of CRC is low, and early screening is hampered by complicated techniques, expensive costs, and the highly invasive nature of CRC. Therefore, many patients are diagnosed at an advanced stage. Because there is currently no effective treatment for CRC, the prognosis of CRC patients is very poor (Carethers and Jung, 2015). Consequently, it is urgent to find more reliable and useful predictive biomarkers for the early identification and diagnosis of CRC.

Many studies have indicated that piRNAs are involved in the process and development of CRC. The high expression level of piR-823 is positively correlated with the proliferation of CRC cells (Yin et al., 2017). piR-823 has been shown to recruit HSF1, a common transcription factor that upregulates heat shock proteins to exert its phosphorylation and transcriptional activity (Yin et al., 2017). This recruitment ability of piR-823 contributes to colon tumorigenesis (Yin et al., 2017). Additionally, CRC patients with high expression levels of piR-823 have a poorer prognosis than patients with low expression levels (Yin et al., 2017). High levels of piR-823 have been associated with poor treatment outcomes in patients with stage II and stage III CRCs. Furthermore, piR-823 was shown to enhance glucose-6-phosphate dehydrogenase (G6PD) expression to promote glucose consumption in CRC cells and downregulate the content of intracellular reactive oxygen species (ROS) by suppressing the ubiquitination of hypoxia-inducible factor-1α (HIF-1α) (Feng et al., 2020). In addition, the level of piR-54265 in CRC tissues was found to be higher than that in non-tumor tissues, and its expression was inversely correlated with the survival of patients with CRC (Mai et al., 2018). piR-54265 binds to PIWIL2 and forms the PIWIL2/STAT3/phosphorylated-SRC complex, thus promoting CRC metastasis and chemoresistance (Mai et al., 2018), suggesting that piR-54265 might be a hopeful therapeutic target for CRC. In another study, the level of piR-54265 in CRC patients decreased sharply after surgical treatment but then increased after tumor recurrence (Mai et al., 2020). Moreover, piR-54265 has shown significant specificity in the serum of patients with CRC (Mai et al., 2020). Therefore, serum piR-54265 holds the potential as a biomarker for monitoring of CRC.

Similarly, piR-1245 is overexpressed in CRC tissues, and regulates CRC cell survival by modulating the expression of tumor suppressor genes (Weng et al., 2018). Patients with high piR-1245 expression had markedly shortened overall survival times (Weng et al., 2018). By establishing a predictive group of piRNAs, previous studies have found that 5 piRNA molecules (Qu et al., 2019), piR-020619/piR-020450 (Wang et al., 2020), or piR-5937/piR-28876 (Vychytilova-Faltejskova et al., 2018) have stronger diagnostic potential when compared with the traditional marker CEA. The diagnostic potential of piRNAs also showed higher sensitivity and specificity. The expression of piR-017724 (Qu et al., 2019) and PIWIL1 (Sun et al., 2017) in serum was positively correlated with the overall survival and progression-free survival, suggesting that piR-017724 and PIWIL1 are independent prognostic factors in CRC. The overexpression of piR-18849 is connected to the degree of tumor differentiation and lymph node metastasis in CRC patients (Yin et al., 2019). Thus, piR-18849 may act as a potential therapeutic target for CRC and as an index to judge patient prognosis. The high piR-24000 expression is notably correlated with the phenotype of invasive CRC, including poor differentiation, distant metastasis, and advanced stage (Iyer et al., 2020). Furthermore, ROC analysis has indicated that there is an observable diagnostic ability of piR-24000 to distinguish CRC patients from healthy subjects (Iyer et al., 2020). Taken together, dysregulation of piRNAs is closely implicated in multiple signaling pathways that regulate the development and progression of CRC, and they could be critical diagnostic and prognostic biomarkers and vital therapeutic targets for CRC (Figures 2, 3). However, the investigation of piRNAs in CRC is preliminary, and the role of piRNAs and their underlying mechanisms require further in-depth study.

### 3.3 PIWI-Interacting RNA and Gastric Cancer

GC is among the top 5 most common malignant tumors worldwide and is the third highest cause of mortality (Bray et al., 2018). The incidence of early gastric cancer has been extremely high, and the radical cure probability of patients with early GC is relatively higher than that of patients with advanced GC (Bray et al., 2018). Patients with advanced GC often have a poor prognosis. Therefore, there is an urgent need for developing new GC markers that can assess the progression of GC and forecast treatment outcomes.

Studies of piRNA profiles have found that piRNAs are abundant in the human stomach (Lin et al., 2019). Transcript analysis of healthy gastric tissues and GC samples identified that nearly half of piRNAs were upregulated in GC samples (Martinez et al., 2016). This implies that piRNAs might impact the pathogenesis of GC. piR-651 is more abundant in GC tissues than in non-cancer tissues, and downregulation of piR-651 inhibits the growth of GC cells (Cheng et al., 2011). The level of piR-823 is reduced in GC cell lines and GC tissues, and overexpression of piR-823 suppresses GC cell growth (Cheng et al., 2012). Experiments in nude mice demonstrated that piR-823 has a tumor-suppressive effect *in vivo* (Cheng et al., 2012). In another study, a ROC curve analysis has shown that the peripheral blood level of piR-823 was a valuable biomarker for differentiating GC patients from healthy controls (Cui et al., 2011). The high PIWIL2 expression was associated with shorter overall survival of GC patients (Wang et al., 2012). PIWIL1 is highly expressed in GC cell lines, and preventing PIWIL1 expression was shown to suppress the malignant behavior of GC cells (Gao et al., 2018). Overall, piRNAs and PIWI proteins could be used as new biomarkers for GC screening, GC diagnosis, and prognosis prediction, and targeted therapy (Figures 2, 3).

### 3.4 PIWI-Interacting RNA and Pancreatic Cancer

Pancreatic cancer is the eighth most prevalent cancer in women and the 10th most common cancer in men (Chen et al., 2016). Pancreatic cancer is a highly malignant digestive tract cancer and is difficult to diagnose and treat. The expression of piR-017061 is downregulated in pancreatic cancer tissues than in normal tissues with a fold change of 2.3 (Müller et al., 2015). piR-017061 attenuates the development and growth of pancreatic cancer cells by cooperating with PIWIL1 to facilitate *EFNA5* mRNA degradation (Xie et al., 2021). These preliminary findings indicated that piR-017061 should be further investigated as a clinical marker of pancreatic cancer.

### 3.5 PIWI-Interacting RNA and Esophageal Cancer

Esophageal carcinoma is the sixth leading cause of death in humans, and its incidence is rapidly rising (Pennathur et al., 2013; Smyth et al., 2017). Overexpression of piR-823 was detected in esophageal cancer tissues, and the levels of piR-823 were positively correlated with the risk of lymph node metastasis (Su et al., 2020). Using ROC curve analysis, piR-823 was identified as a valuable biomarker for differentiating esophageal cancer from normal controls (Su et al., 2020). In addition, the expression of piRNA-823 and DNMT3B were positively associated with each other, indicating that piRNA-823 might play an oncogenic function in esophageal cancer by inducing aberrant DNA methylation via DNMT3B (Su et al., 2020). A higher amount of PIWIL1 protein expression in the cytoplasm of esophageal cancer cells is correlated to higher histological grade, advanced tumor stage, and poorer overall survival (He et al., 2009). More comprehensive research is required to understand the specific mechanisms of piR-823 in esophageal cancer.

### 3.6 PIWI-Interacting RNA and Biliary Tract Cancer

Biliary tract cancer arises from epithelial cells lining the biliary tract. Plasma exosomal piRNAs can be either significantly upregulated or downregulated in these patients (Gu et al., 2020). The levels of piR-10506469 were significantly increased in plasma exosomes from cholangiocarcinoma malign cholangiocarcinoma or gallbladder carcinoma patients compared with healthy individuals (Gu et al., 2020). Furthermore, the expression of piR-10506469 and piR-20548188 were significantly reduced after surgery (Gu et al., 2020). Thus, these piRNAs might serve as potential biomarkers of cholangiocarcinoma and gallbladder carcinoma.

## 4 Therapeutic Approaches Using PIWI-Interacting RNAs

The potential of piRNAs to affect numerous downstream pathways can bring a significant impact on the molecular and functional landscape of cancer cells, promoting attempts to create future therapies that specifically target piRNAs (Jacovetti et al., 2021). Numerous preclinical research employing piRNA-based therapeutic compounds has already demonstrated outstanding results in terms of the capacity of piRNAs to influence the malignant features of HCC, CRC and GC cells (Cheng et al., 2012; Law et al., 2013; Mai et al., 2018) (Figure 2). The silencing of piR-Hep1 with a locked nucleic acid inhibitor inhibited cell viability, motility, and invasiveness in HCC cells (Law et al., 2013). In CRC cells, piR-54265 acts as an oncogenic piRNA, and overexpression of piR-54265 activates STAT3 signaling, consequently enhancing the proliferation, metastasis, and chemoresistance of CRC cells (Mai et al., 2018). Knockdown of piR-54265 using shRNA was associated with the inhibition of invasive ability and colony-forming capacity as well as attenuation of tumor growth in nude mice (Mai et al., 2018). Treatment with a specific chemically modified piR-54265 inhibitor significantly suppressed the growth and metastasis of implanted tumors in mice, and improved the sensitivity of CRC cells to 5-FU *in vivo* (Mai et al., 2018). These findings suggest that piR-Hep1 and piR-54265 could be druggable targets for the effective treatment of digestive cancers, and that combined chemotherapy with a piR-54265 inhibitor could be a viable future treatment option for CRC (Figure 3).

On the other hand, the restoration of tumor-suppressive piRNA could be considered another tool to achieve significant anti-tumor effects. For instance, lentiviral vector-mediated overexpression of piR-36712 in breast cancer cells suppressed malignant phenotypes and had a synergistic anti-tumor effect when combined with chemotherapy agents (Tan et al., 2019). Moreover, piR-823 mimics could significantly inhibit the growth of GC cells both *in vitro* and *in vivo* (Cheng et al., 2012). This observation suggests that piR-823 is a possible therapeutic target in digestive cancers (Figure 3).

## 5 Future Perspectives

piRNAs have gradually attracted increasing attention since they were first discovered in animal germ cells in 2006. Although several studies have demonstrated a relationship between piRNAs and cancer biology, their roles and the respective regulatory mechanisms require further exploration. The following questions remain open for investigation:

1) How to precisely quantify piRNAs? Different piRNA expressions have been reported in cancer and adjacent normal tissues. However, the molecular features of adjacent normal tissues might be similar to that of cancer tissues (Krishnan and Damaraju, 2018). As a result, using surrounding normal tissues as a reference might lead to erroneous interpretation of piRNA expression. Normal tissues collected from healthy individuals may serve as a better control for comparison with tumor tissues (Krishnan and Damaraju, 2018).
2) How are piRNA transcripts generated in human cancer cells? HSP83/Shu is believed to play a role in the PIWI loading step, and HSP90 and its co-chaperone FKBP6 are required for the secondary piRNA biogenesis (Ishizu et al., 2012). However, most of our knowledge comes from Drosophila germline cells (Wu et al., 2020), and the exact mechanisms underlying piRNA biogenesis in human tumor cells remain largely unknown.
3) What are the mechanisms by which piRNAs exert their functions? Currently, the underlying mechanisms that account for the biological functions of piRNAs in tumor cells are still unclear. Upregulation of PIWI protein was a frequent event in many tumor types (Dong et al., 2021). Even in the absence of piRNAs, PIWI could interact with other molecules to induce tumorigenesis, cancer metastasis, and chemoresistance through piRNA-independent pathways (Dong et al., 2021). piRNA-interacting partners can be detected by high-throughput experimental approaches (Huang et al., 2021).
4) Do genetic variants alter the functions of the mature piRNAs, leading to their deregulation and the carcinogenic process? Single-nucleotide polymorphisms (SNPs) and insertion-deletion (INDELs) are of particular clinical importance due to their ability to impair gene functions (Karki et al., 2015). Some SNP variants in piRNA sequences have been associated with an increased risk of cancer development (Fu et al., 2015). Thus, it would be crucial to explore the effects of these genetic variations on piRNA functions and the development of digestive system cancers.
5) What are the roles of piRNAs in cancer stemness? The emerging roles of piRNAs in mediating cancer stem cell (CSC)-like properties have been observed (Su et al., 2021). It has been demonstrated that piR-823 was significantly upregulated in the ALDH-positive breast CSCs, and piR-823 confers stem-like properties to breast cancer cells by activating the Wnt signaling pathway (Ding et al., 2021). In clear cell renal carcinoma cells, piR-31115 induces epithelial-mesenchymal transition (EMT) via decreasing E-cadherin expression and increasing mesenchymal markers (Vimentin and Snail) (Du et al., 2021). These results suggest that the expression of certain piRNAs is required for the initiation and maintenance of CSCs, and the roles of piRNAs in gastrointestinal CSCs deserve further investigation.

## 6 Conclusion

At present, the approaches for the early diagnosis of major digestive system cancers are limited, and the prognosis of patients with digestive system cancers is still poor. Therefore, there is an urgent need to find more accurate and convenient clinical biomarkers that can assist in the diagnosis and treatment of these diseases. Growing evidence suggests that some individual piRNAs (such as piR-823 and piR-54265) modulate the occurrence, progression, and chemoresistance in multiple digestive cancers (such as HCC, CRC and GC) (Figure 2). However, the roles of dysregulated PIWI-piRNA pathway in digestive cancers have not been thoroughly investigated. Additional in-depth research will help to clarify the specific mechanisms by which piRNAs affect digestive system cancers. In conclusion, piRNAs represent new candidate diagnostic/prognostic biomarkers for digestive system cancers, as well as possible targets for future cancer therapy (Figure 3).
